# Bi-directional Acceleration of Alcohol Use and Opioid Use Disorder

**Published:** 2019-10-18

**Authors:** Wenfei Huang, Erika P. Penaherrera, Danaika F. Desir, Dominick L. Gamarro, Jessica Cottrell, Tinchun Chu, Sulie L. Chang

**Affiliations:** 1 Department of Biological Sciences, Seton Hall University, South Orange, NJ, USA; 2 Institute of NeuroImmune Pharmacology, Seton Hall University, South Orange, NJ, USA

**Keywords:** alcohol abuse, alcohol use disorder, ingenuity pathway analysis, opioid dependence, opioid use disorder

## Abstract

Alcohol is the most widely used addictive substance. Severe alcohol abuse is diagnosed as “alcohol use disorder” (AUD). A common and harmful drinking pattern is binge drinking that elevates a person’s blood alcohol concentration to ≥ 0.08%. Such drinking may be an early indicator of AUD. Opioid misuse and dependence have become worldwide crises. Patterned consumption of various opioids can develop into opioid use disorder (OUD). An intertwined epidemic exists between opioid abuse, alcohol addiction, and binge drinking. Currently, studies on the interaction of AUD and OUD are limited and the underlying mechanisms linking these disorders remains unclear. We reviewed studies on AUD and OUD and utilized Ingenuity Pathway Analysis (IPA) to identify mechanisms of AUD and OUD interaction and potential gene targets for therapeutic agents. According to IPA Canonical Pathways Analysis, Gamma-aminobutyric Acid (GABA) Receptor Signaling, Neuroinflammation Signaling Pathway, Opioid Signaling Pathway and Dopamine-DARPP32 Feedback in cAMP Signaling are potential contributors to the interaction of AUD and OUD.

## 1. Introduction

### 1.1. Binge drinking and alcohol use disorders

Alcohol is the most widely used addictive substance in the world. The effects of drinking depend on the volume consumed, the concentration of ethanol by volume, and the drinking pattern. [1–4]. Alcohol consumption volume is the most commonly used measurement in alcohol research. The overall volmne of alcohol consumed was found to be related to various alcohol-related disease, such as alcohol dependence, unipolar major depression and cirrhosis of the liver [5]. Based on the US Natural Alcohol Survey, Relative Risk (RR) of Mortality in heavy drinkers with ≥ 8 drinks on average was 70% higher than non-heavy drinkers [4]. Drinking patterns are defined in terms of frequency of drinking. One especially harmful pattern is binge drinking, which is defined by the National Institute on Alcohol Abuse and Alcoholism (NIAAA) as drinking that brings a person’s blood alcohol concentration (BAC) to ≥ 0.08 g % after consumption of 5 or more drinks for men or 4 or more drinks for women in about 2 hours [6]. Binge drinking is the most common and deadly pattern of excessive alcohol use in the United States [7–9], being associated with health risks such as alcohol poisoning, alcohol dependence, cancer, and other chronic diseases [10].

Alcohol abuse that becomes severe is called “alcohol use disorder” (AUD) [11]. This chronic relapsing brain disease is characterized by compulsive alcohol use, loss of control over alcohol intake, and a negative emotional state when not drinking [11]. According to the NIAAA, 16 million people in the United States have AUD, of which approximately twice as many are men (9.8 million *vs.* 5.3 million) [11]. The disorder includes alcohol abuse and alcohol dependence. Alcohol abuse is a pattern of regular alcohol intake that prevents performance of daily tasks. A person with alcohol abuse problems tends to drink too much even in situation where such drinking is physically dangerous to them. Alcohol dependence is a classic addiction: the inability to quit drinking. Binge drinking is part of both disorders.

### 1.2. Opioid use disorders

Opioids are natural or synthetic chemicals that interact with opioid receptors on the cell surface and reduce feelings of pain. Opioids include the illegal drug heroin, synthetic opioids such as fentanyl, and pain relievers available legally by prescription, such as morphine, oxycodone, hydrocodone, and codeine [12].

Opioids exert their effects by binding to the one or all three (mu, delta, kappa) opioid receptors in an agonistic, antagonistic fashion [13]. Prescription opioids are meant to be used to treat acute pain (such as when recovering from injury or surgery), chronic pain, active-phase cancer treatment, palliative care, and end-of-life care. Many people rely on prescription opioids, under the care of a physician, to help manage their conditions [14]. Opioids also create significant positive reinforcement, increasing the odds that people will continue using them despite negative consequences, leading to abuse or addiction [13]. Patterned consumption causes opioid use disorder (OUD), which if not interrupted is a lifelong disorder with serious consequences such as significant social and physical functional impairment [15, 16], disability, and death [17]. Even if a patient is weaned from the drug, the risk of relapse is high; and patients may well manifest all the features of addiction. OUD involves opioid addiction and opioid dependence. Opioid addiction is characterized by a pathologically compulsive urge to use opioid drugs [18]. Opioid dependence results from taking opioids over a long period of time, such that patients show physical and psychological symptoms of withdrawal when stop taking the drug [19]. Opioid misuse and dependence have increased rapidly worldwide. In 2017, more than 70,200 Americans died from drug overdoses, mostly of opioids, a two-fold annual increase in a decade [20].

### 1.3. Comorbidity of AUD and OUD

During the last decade, intertwined epidemics of alcohol use and opioid use have emerged [21]. Among 3.1 million patients with OUD and related conditions, AUD is diagnosed 8.4 times more frequently than in patients without OUD, and binge drinking is 5.0 times more common in patients with OUD. According to the report on alcohol and drug use from the Michigan Department of Health and Human Services, among youths in 2011, 28.0% of binge drinkers reported using painkillers (including oxycontin, codeine, and Percocet), whereas only 7.0% of nondrinkers and 15.6% of non-binge drinkers took opioids [22].

There are bi-directional interaction between Alcohol Use Disorder (AUD) and Opioid Use Disorder (OUD). AUD has been found to be associated with increased likelihood of misusing prescription drugs [23, 24]. OUD including opioid misuse may increase the risk of co-occurring AUD [23, 25]. Besides, Comorbid OUD and AUD interfere with medication adherence and carry high morbidity and mortality rates [26]. Clinical and preclinical studies have confirmed interactions of AUD and OUD. For example, Kao analyzed the medical records of 4,143 patients using intravenous patient-control opioid analgesia after abdominal surgery and reported that frequent alcohol drinking was associated with greater opioid demand for control of pain and decreased nausea and vomiting [27]. In preclinical studies, cross-tolerance between alcohol and opioid abuse has been proved [28]. Campbell and colleagues reported that acute administration of alcohol to animals can produce a moderate anti-nociceptive effect through interaction in the central nervous system (CNS) [29].

The mechanism underlying the interaction of AUD and OUD is still a puzzle. In this project, we used a bioinformatics tool to explore the genetic basis and molecular mechanism underlying the correlation between alcohol, and opioid use. Understanding the genetic bases of AUD and OUD will encourage integration of genetic studies into the process of drug administration, as well as improve tailoring patients’ medication according to their medical histories and genetic risk factors [30].

### 1.4. Ingenuity pathway analysis to predict potential gene network

Ingenuity Pathway Analysis (IPA) is a software application based on computational algorithms that analyzes the functional connectivity of the genes from information in the comprehensive, manually curated Ingenuity Knowledge Base (Qiagen Bioinformatics, Redwood City, CA). IPA helps identify connections between genotypes, phenotypes, diseases, drugs, and drug trials to pinpoint disease-causing mutations, new biomarkers, or the right treatment for a patient [31]. IPA and other bioinformatics tools have been used in basic and clinical studies, in planning and justifying new research, in identifying elements of current studies and unknown questions, systematic analysis of meta-data, and more.

## 2. Methods

We first conducted a literature search in PubMed, and Science Direct using the terms “alcohol use disorder,” “binge drinking,” “alcohol abuse,” “opioid use disorder,” and “opioid abuse.” Few studies report on direct interaction between AUD and OUD, and only 7 relevant papers were identified and reviewed.

With the goal of identifying genes and pathways important to AUD and OUD, a comprehensive gene network analysis was conducted using IPA. The genes involved in AUD or OUD were identified using the search bar tool in IPA based on IPA Knowledgebase: The gene set related to AUD were identified using the defined terms representing AUD in IPA “alcohol abuse,” “alcoholism,” “alcohol use disorder,” [11]; and the gene set related to OUD were identified using the terms “opioid-related disorders”, “opioid use disorders”, “opioid dependence”, “opioid addiction”, which represented OUD [14]. The genes reported to be associated with any of these conditions were made evident by overlapping the two sets of genes in My Pathways tool. Relations between the genes and defined the diseases were mapped based on 87 studies from IPA Knowledgebase supporting the correlation of the genes with the diseases. The 87 literatures and their relevance to these genes related to AUD and OUD were reviewed and tabulated in Tables 1–6.

To analyze the functional connectivity of the genes, the gene set was imported into IPA for canonical pathway analysis through accessing pathway enrichment based on the associations documented in the IPA database. Canonical pathways were scored by calculating a ratio of the number of genes that map to the pathway. The pathways were scored on the basis of the negative base_10_ logarithm of the p value obtained using Fisher’s exact test (−log_10_ [*p* value]) [32–34].

## 3. Results

Among the 235 genes related to AUD and the 131 genes related OUD, there were 65 genes related to both disorders. The relations of the 65 genes to AUD (defined tenns “AUD” (Table 1) “alcohol abuse” (Table 2) “alcohol dependence” (Table 3), and “OUD: (defined tenns “OUD” (Table 4), “opioid dependence” (Table 5) and “opioid addiction” (Table 6) were reviewed. The doubly active genes were analyzed further using IPA core analysis, predicting pathways possibly involved in the interaction. Highly enriched canonical pathways were evaluated by p values from Fisher’s exact test. The top ten canonical pathways were Gamma-aminobutyric Acid (GABA) Receptor Signaling, Neuroinflammation Signaling, Opioid Signaling, G-protein Coupled Receptor Signalling, Dopamine-DARPP32 Feedback in cAMP Signaling, Gαi Signalling, Circadian Rhythm Signalling, cAMP-mediated signalling, nNOS Signaling in Neurons, Glutamate Receptor Signalling.

## 4. Discussion

During the last decade, intertwined epidemics of opioid abuse, binge drinking, and alcohol addiction have emerged [21]. The co-existence of these three medical processes could have led to death associated with their comorbidity [26]. Unfortunately, only a few studies have explored the interaction between and among these disorders, and the mechanisms underlying it remain unclear. In this review, we investigated the connection between AUD and OUD, utilizing IPA software application.

**Figure 1:**
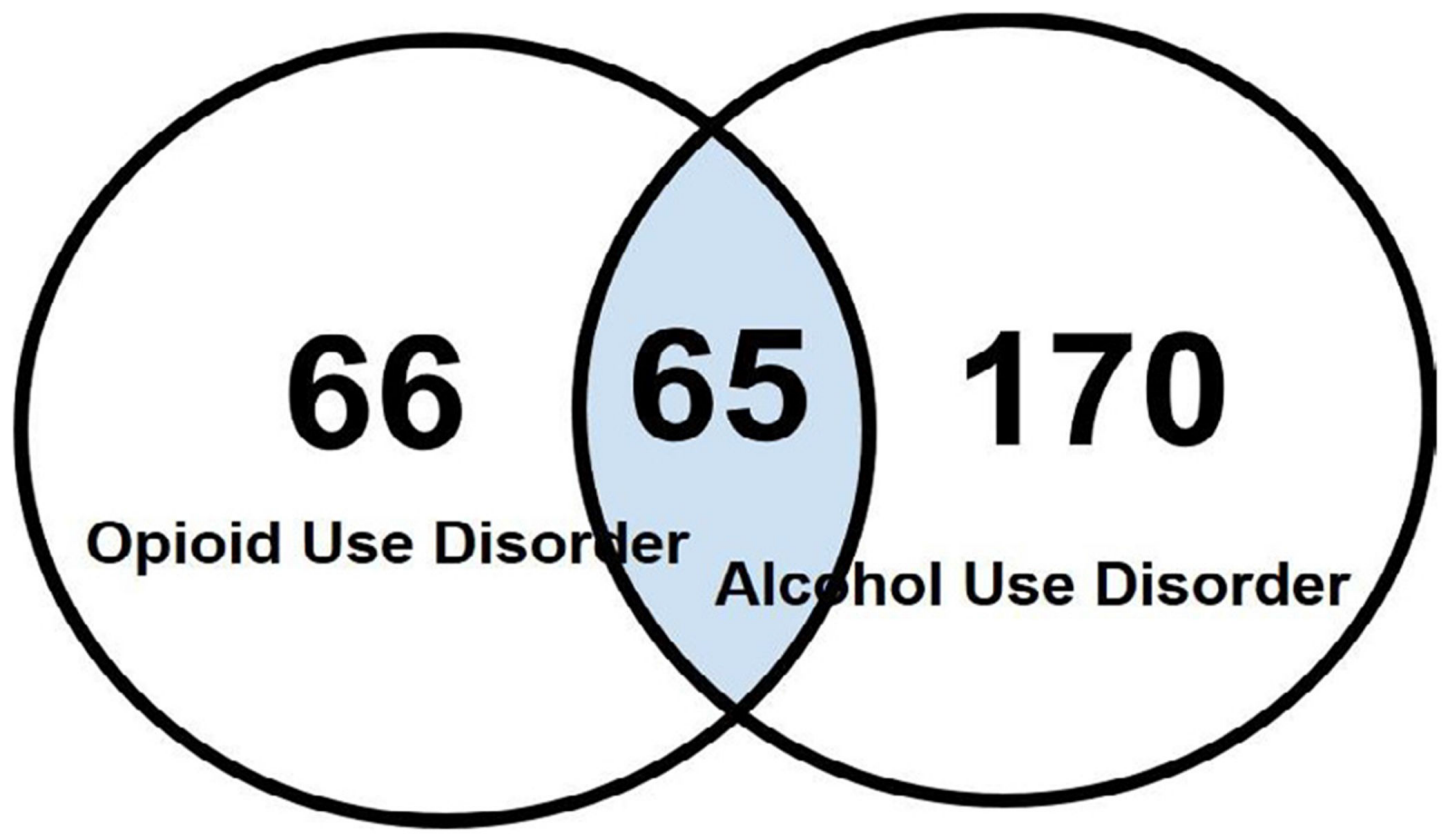
Genes that might contribute to interaction of alcohol abuse/AUD and OUD, as identified using IPA.

**Figure 2:**
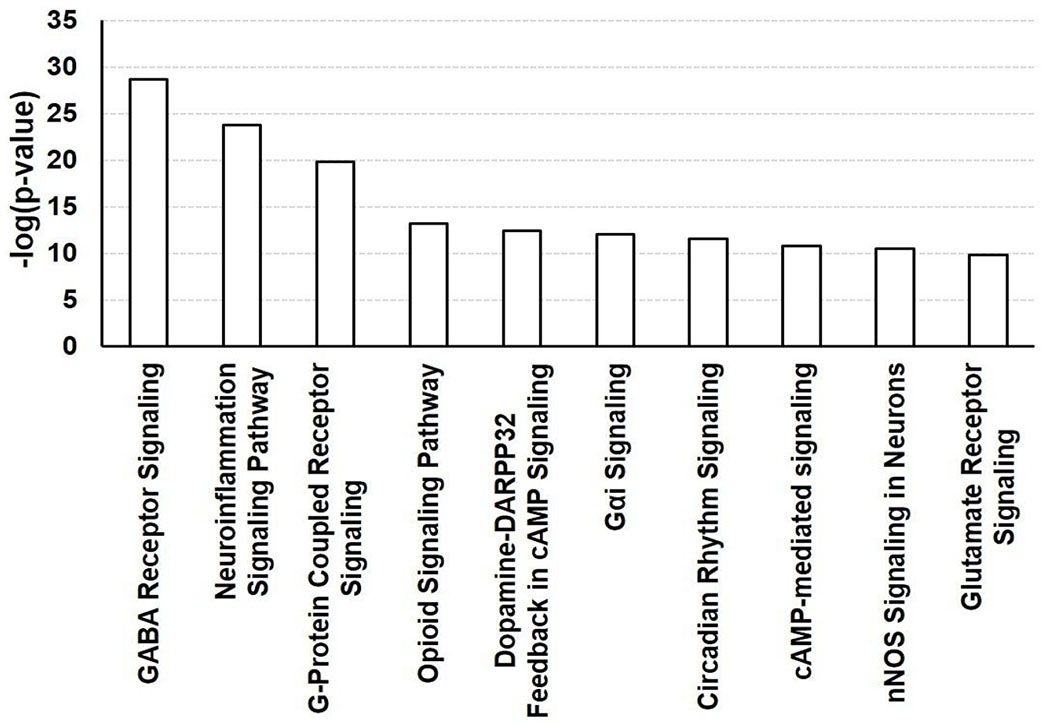
Canonical pathway analysis of the genes related to AUD and OUD, as analyzed using IPA core analysis. The gene set was imported into IPA for canonical pathway analysis. Such pathways were scored by analyzing the ratio of the number of genes that map to the pathway. The pathways are presented according to the negative base_10_ logarithm of the p value obtained using the Fisher exact test.

**Figure 3:**
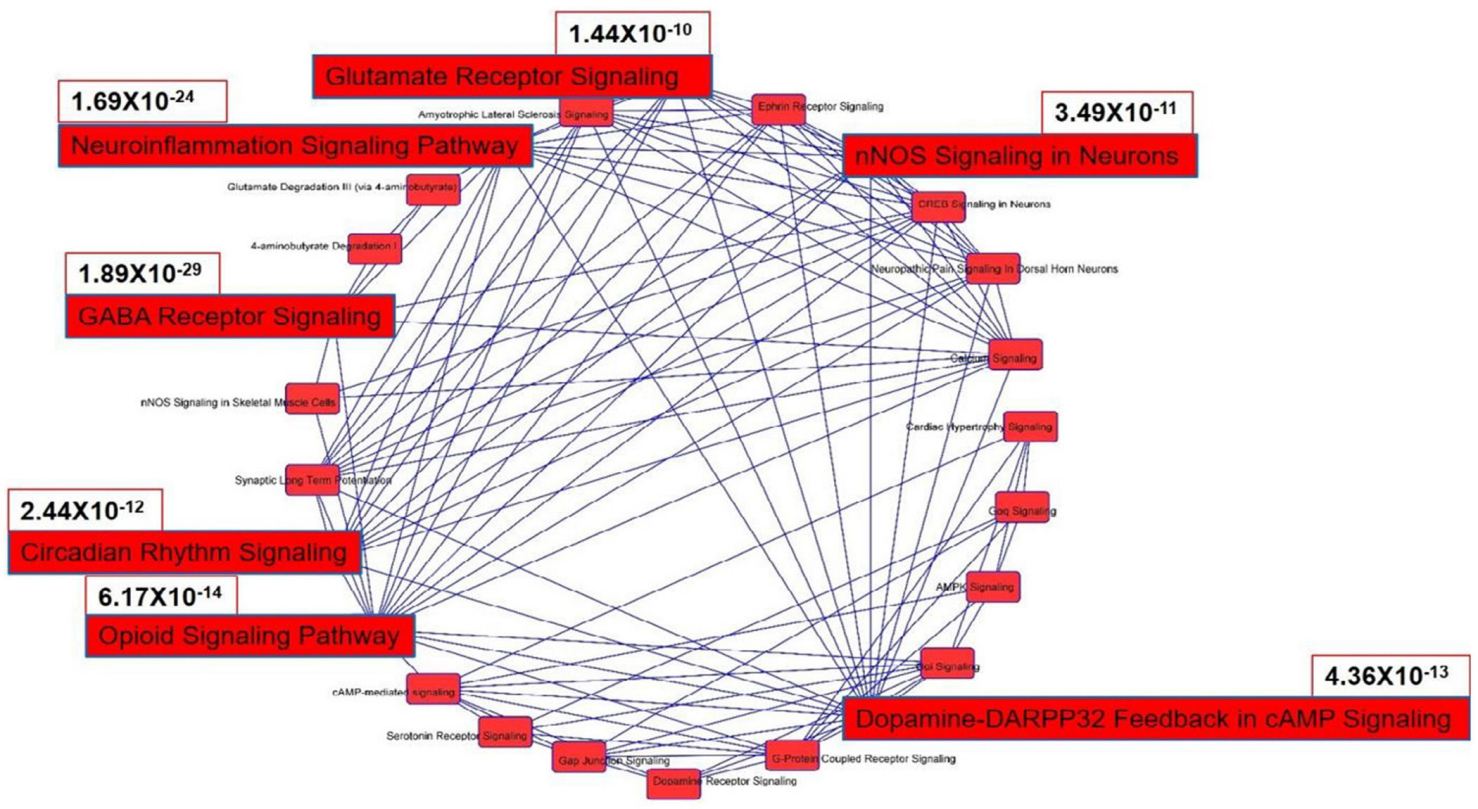
Overlapping of the canonical pathways predicted to contribute to interaction of AUD and OUD. Interactions between predicted pathways were analysed based on overlap of the genes in the canonical pathways. Seven top pathways are highlighted: Gamma-aminobutyric Acid (GABA) Receptor Signaling, Neuroinflammation Signaling, Opioid Signaling, dopamine-DARPP32 feedback in cAMP signaling, Circadian Rhythm Signalling, nNOS Signaling in Neurons and Glutamate Receptor Signalling. The P values of the 7 pathways are labelled beside the pathway.

**Figure 4:**
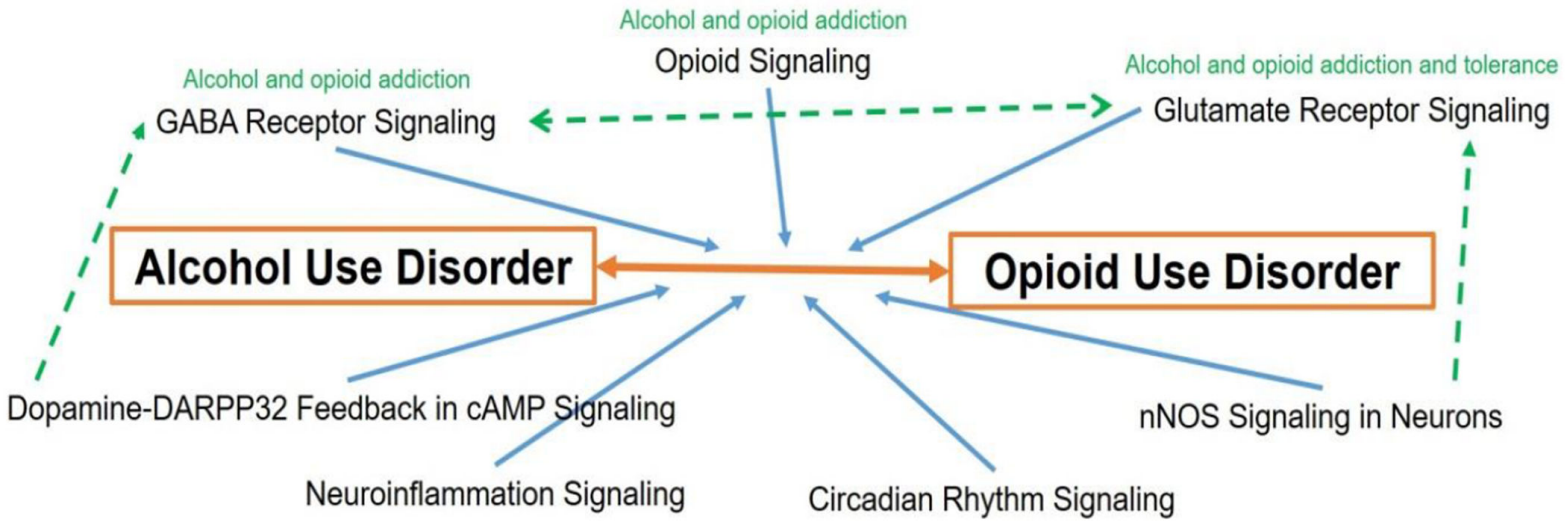
Predicted contribution of signalling pathways to interaction of AUD and OUD. Orange arrow refers to the interaction of AUD and OUD. Blue arrow refers to predicted involvement of the pathways in the interaction of AUD and OUD. Green dash arrows and green notes refer to correlations between pathways and disorder symptoms supported by literatures.

Our analysis found that 65 genes are associated with both AUD and OUD, a finding supported by 87 literatures retrieved from IPA Knowledgebase, including clinical studies utilizing corresponding inhibitors (Figure 1 and Table 1). Canonical pathway analysis of these genes predicted pathways likely contributing to the interaction (Figures 2 and 3). Although some of the inhibitors used in the reported clinical studies act on a specific family of genes, which may result in redundant correlations. Canonical Pathways Analysis can help identify relevant genes efficiently. Among the Top 10 pathways predicted to contribute to the interaction of AUD and OUD, the role of Gamma-Aminobutyric Acid (GABA) Receptor Signaling, Nemoinflammation Signaling, Opioid Signalling and Glutamate Receptor Signalling were supported by studies; involvement of Dopamine-DARPP32 Feedback in cAmp Signaling, Circadian Rhythm Signalling, nNOS Signaling in Neurons were understudied; G-protein Coupled Receptor Signalling, Gαi Signalling and cAMP-mediated signalling referred to a general group of pathways highlighting involvement of G-protein, G-α subunit or cAMP, respectively. Thus current findings on the involvement in AUD and OUD of file seven pathways (GABA Receptor Signaling, Neuroinflammation Signaling, Opioid Signalling and Glutamate Receptor Signalling, Dopamine-DARPP32 Feedback in cAMP signaling. Circadian Rhythm Signalling, nNOS Signaling in Neurons) were highlighted (Figure 4).

Gamma-aminobutyric Acid (GABA) Receptor Signaling, Neuroinflammation Signaling, Opioid Signaling, dopamine-DARPP32 feedback in cAMP signaling. Circadian Rhythm Signalling, nNOS Signaling in Neurons and Glutamate Receptor Signalling are predicted to be involved in AUD and OUD interaction in IPA. Literatures reported that GABA Receptor Signalling and Opioid Signalling have been reported to be related to alcohol and opioid addiction; Glutamate Receptor Signalling has been reported to be related to alcohol and opioid addiction and tolerance. It is found that Dopamine-DARPP32 feedback in cAMP signalling cross-talks with GABA Receptor Signaling and that nNOS Signaling in Neurons has cross-talk with Glutamate Receptor Signalling. In addition, cross-talk and regulation between GABA Receptor Signaling and Glutamate Receptor Signalling have been reported. Involvement of Neuroinflammation Signaling and Circadian Rhythm Signalling is unclear.

Alcohol exerts effects on the reward system through both the opioid and dopaminergic systems [35]. GABA Receptor Signaling is involved in both alcohol and opioid addiction [36–38]. Specifically, opioids inhibit GABA-mediated neurotransmission [39], and alcohol exerts its rewarding action through GABA receptors [40, 41]. The GABA receptor agonist baclofen, which inhibits ethanol consumption, decreases alcohol-induced craving and withdrawal symptoms [42–44]. Moreover, a recent study reported that alcohol mediates non-canonical GABA synthesis through aldehyde dehydrogenase 1a1 (ALDH1a1) in midbrain dopaminergic neurons [45].

Glutamate is the most abundant excitatory neurotransmitter in the mammalian nervous system, and acts via glutamate receptors. Both alcohol and mu-opioid are well-known to affect glutamate receptor function. Alcohol is known to inhibit glutamate, particularly at the N-methyl-d-aspartate (NMDA) glutamate receptor [46–48]. And NMDA glutamate receptors associate with mu-opioid and could contribute to opioid tolerance [49–51]. On the other hand, as the excitatory and inhibitory neurotransmitter systems, glutamate signalling and GABA signalling cross-talked and coordinated to maintain brain function [52, 53]. Thus Glutamate Receptor Signalling could be involved in interaction of AUD and OUD directly and indirectly *via* GABA Receptor Signalling.

Opioid receptor genes have been studied frequently in relation to both alcohol and opioid addiction [54–58]. It is well known that opioid receptors, especially the mu-opioid receptor (MOR), play an important role in the rewarding properties of ethanol [59, 60]. MOR antagonists are mainstays of the treatment of alcohol withdrawal syndrome, as well as OUD [61, 62]. The opioid antagonist naltrexone reduces the desire to drink and the amount of alcohol consumed by alcohol-dependent individuals, implying that it would be an effective treatment for alcohol-dependent subjects [63, 64]. MOR-encoding gene *OPRM1* polymorphisms are found to be associated with alcoholism treatment and risk [65]. Adolescents with the A/G or G/G genotypes of A118G are three times more likely to have an AUD than are those with the A/A genotype [66, 67]. The link between *Oprm1* polymorphism and alcohol dependence may be explained by different physiological responses to alcohol by persons with the various genotypes. People with the 118G variant have a stronger association between their desire to drink and subsequent drinking than people lacking the variant, an effect attenuated by naltrexone [68]. It is less clear how excessive ethanol consumption affects opioid receptors and downstream signaling. He and Whistler reported that chronic ethanol drinking alters the ability of MOR to endocytose in response to opioid peptides and consequently promotes tolerance to the effects of opioids [69]. And cross-tolerance between alcohol intake and morphine treatment have been proved [28, 69].

Perhaps surprisingly, the inflammation and immune response also is associated with the rewards of alcohol and opioid addiction [34]. Neuroinflammation in the CNS is shared by the response to chronic pain and opioids and contributes to pain sensitization and negative affect [70]. Recent evidence confirms that glial activation and upregulation of inflammatory mediators in the CNS play pivotal roles in neuropathic pain and opioid tolerance. Blocking pro-inflammatory glial activation might block the elevation of dopamine induced by opioid receptor activity. Hutchinson and associates found evidence that Toll-like receptors (TLRs), a class of innate immune receptors, interact with opioids and glial cells, contributing to opioid reward behaviors [71, 72]. Chang et al. [73] created binge exposure to ethanol-enhanced morphine anti-nociception in B6 mice, confirming the role of inflammation [73].

Cyclic AMP-regulated phosphoprotein (DARPP-32) is a key actor of this integration in the GABAergic medium-size spiny neurons, particularly in response to dopamine [74, 75]. Increased cAMP activates cAMP-dependent protein kinase (PKA). Once active, PKA phosphorylates DARPP32 and induces phosphorylation cascades and the dopamine neurotransmission system. DARPP32 act by activating calcium-dependent proteins such as calmodulin and calcineurin [74, 76]. A key role of GABA-mediated dopamine signaling in alcohol and opioid action has been well studied, yet the direct role of dopamine-DARPP32 feedback is unclear.

Circadian rhythm is a roughly 24-hour periodicity in the physiological processes in various organisms, which is endogenously maintained by a series of molecular mechanisms. The circadian rhythm, however, can be moderated by external environment, such as light and temperature. Limited studies have reported effects of alcohol and opioid use on circadian rhythm. Baird et al reported that circadian rhythm of body temperature changed following alcohol treatment [77, 78]. Binge drinking and chronic alcohol use can disrupt circadian rhythms and induce the addictive behaviour associated with AUD [79, 80]. The circadian rhythm is also reported to be affected by acute morphine treatment in rat [81], yet the role of circadian rhythm in OUD remained unclear.

Nitric oxide (NO) is formed endogenously by a family of enzymes known as NO synthases (NOS) Neuronal nitric oxide synthase (nNOS) is a family of enzymes producing Nitric oxide (NO), expressed in specific neurons of the Central Nervous System (CNS) [82]. nNOS is stimulated by free cytosolic Ca^2+^ through interaction with CALM and Calcineurin. In the brain, NO synthesis is regulated through stimulation of NMDAR by glutamate [83, 84]. NOS expression increased in alcoholic brain [85, 86], and had a protective effect against alcohol toxicity [87] Meanwhile, morphine treatment is known to produce nNOS in a Ca^2+^-dependent manner [88]. Direct effect of nNOS in interaction of AUD and OUD is unknown.

## 5. Conclusion

In summary, this review studied AUD and OUD, and predicts potential targets in AUD and OUD interaction based on a bioinformatics tool, IPA. Multiple pathways could contribute to the interaction. According to IPA Canonical Pathways Analysis, GABA Receptor Signaling, Neuroinflammation Signaling Pathway, Opioid Signaling Pathway and Dopamine-DARPP32 Feedback in cAMP Signaling are potential pathways contributing to interaction of AUD and OUD. Our review provides a possible strategy for studying disease interaction and mining drug targets.

## Figures and Tables

**Table 1:** Genes reported related to Alcohol Use Disorder (AUD).

Gene symbol	Finding	Citations
*ABAT, ALDH5A1*	Valproic acid, an inhibitor of *ABAT* and *ALDH5A1*, is being used in Phase IV clinical trials to create lorazepam and valproate drugs for the prevention of alcohol use disorder	[89]
*CACNA2D1, CACNA2D2*	Pregabalin, a binder of human CACNA2D1 and CNA2D2 proteins, is in Phase III as a treatment for AUD	[90]
*GABRA1, GABRA3, GABRA4, GABRA5*	Pregnenolone, an inhibitor of human GABRA1, GABRA3, GABRA4, and GABRA5 proteins, is in Phase IV as a treatment for alcohol use disorder	[91]
	Diazepam, a modulator of human GABRA1, GABRA3, GABRA4, and GABRA5 proteins, is in Phase IV with baclofen as a treatment for alcohol use disorder	[92]
	Lorazepam, a modulator of human GABRA1, GABRA3, GABRA4, and GABRA5 proteins, is in Phase IV with valproate [valproic acid] to prevent alcohol use disorder	[93–94]
	Lorazepam, a modulator of human GABRA1, GABRA3, GABRA4, and GABRA5 proteins, is in Phase IV with carbamazepine as a treatment for alcoholism	[94–95]
*GABRB2*	Lorazepam, a modulator of human GABRB2 proteins, is in Phase IV with valproate [valproic acid] to prevent alcohol use disorder	[93–94]
	Midazolam, a modulator of human GABRB2 proteins, is in Phase IV as supportive care for alcoholism	[96]
	Propofol, a modulator of human GABRB2 protein, is in Phase IV as supportive care for alcoholism	[96]
*GABRG1, GABRG2*	Chlordiazepoxide, a modulator of human GABRG1 and GABRG2 proteins, is in Phase III as a treatment for alcoholism	[97]
	Lorazepam, a modulator of human GABRA6, GABRB1, GABRB2, GABRB3, GABRE, GABRG1, GABRG2, GABRG3, and GABRP proteins, is in Phase IV with valproate [valproic acid] to prevent alcohol use disorder	[93–94]
	Lorazepam, a modulator of human GABRG1 and GABRG2 proteins, is in Phase IV with carbamazepine as a treatment for alcoholism	[94–95]
	Midazolam, a modulator of human GABRG1 and GABRG2, proteins, is in Phase IV as supportive care for alcoholism	[96]
*GABRA6, GABRB1, GABRB2, GABRB3, GABRE, GABRG3, GABRP*	Lorazepam, a modulator of human GABRA6, GABRB1, GABRB2, GABRB3, GABRE, GABRG1, GABRG2, GABRG3, and GABRP proteins, is in Phase IV with valproate [valproic acid] to prevent alcohol use disorder	[93–94]
	Lorazepam, a modulator of human GABRA6, GABRB1, GABRB2, GABRB3, GABRE, GABRG1, GABRG2, GABRG3, and GABRP proteins, is in Phase IV with carbamazepine as a treatment for alcoholism	[94–95]
	Midazolam, a modulator of human GABRA6, GABRB1, GABRB2, GABRB3, GABRE, GABRG1, GABRG2, GABRG3, and GABRP proteins, is in Phase IV as supportive care for alcoholism	[96]
*GAD2*	Valproic acid, an inhibitor of human GAD2 protein, is in Phase II/III as a treatment for alcoholism	[98]
*GRIN1, GRIN2B, GRIN2A, GRIN2C, GRIN2D, GRIN3A, GRIN3B*	Memantine, an antagonist of human GRIN1, GRIN2A, GRIN2B, GRIN2C, GRIN2D, GRIN3A, GRIN3B proteins, is a potential treatment for alcoholism	[99]
*OPRD1, OPRM1, OPRK1, SIGMAR1*	Naltrexone, an antagonist of human OPRD1, OPRM1, OPRK1, and SIGMAR1 protein, is in Phase III as a component of a treatment for AUD and alcohol abuse	[100]
	Naltrexone, an antagonist of human OPRD1, OPRM1, OPRK1, and SIGMAR1 proteins, has been approved as a treatment for alcoholism	[94, 101]
*SLC6A2, SLC6A3*	Methylphenidate, an inhibitor of human SLC6A2 and SLC6A3 proteins, is in Phase IV as a treatment for alcoholism	[102]
	Bupropion, an inhibitor of human SLC6A2 and SLC6A3 proteins, is in Phase IV as a treatment for alcoholism	[103]
*SLC6A4*	Bupropion, an inhibitor of human SLC6A4 protein, is in Phase IV as a treatment for alcoholism	[103]
	Mutant human *SERT* [*SLC6A4*] gene (unspecified DNA mutation) is associated with alcoholism	[104]
	Sertraline, an inhibitor of human SLC6A4 protein, is in Phase IV as a treatment for alcoholism	[105–106]
	Escitalopram, an inhibitor of human SLC6A4 protein, is in Phase IV with Ebixa [memantine] as a treatment for alcoholism	[107]

**Table 2:** Genes reported related to alcohol abuse.

Gene symbol	Finding	Citations
*ADRA1A, ADRA1B, ADRA1D, ADRA2A,*	Quetiapine and risperidone, both inhibitors of ADRA1A, ADRA1B, ADRA1D, and ADRA2A proteins in humans, are being use in Phase IV clinical trials for the treatment of alcohol abuse	[108–110]
	Aripiprazole, an inhibitor of ADRA1A, ADRA1B, ADRA1D, and ADRA2A proteins in humans, is being use in a Phase III clinical trial for alcohol abuse treatment	[111]
*ADRA2B, ADRA2C*	Quetiapine and risperidone, both inhibitors of ADRA1A, ADRA1B, ADRA1D, ADRA2A, ADRA2B, and ADRA2C proteins in humans, are being used in Phase IV clinical trials for the treatment of alcohol abuse	[108–110]
*CA1, CA3, CA5A, CA5B, CA6, CA9*	Topiramate, an inhibitor of human CA1 protein, is in Phase IV as a part of the combination drug quetiapine and topiramate as a treatment for alcohol abuse. It also is being used alone in a Phase IV clinical trial as treatment for alcohol dependence	[110, 112–113]
*CA2, CA4*	Trokendi XR [topiramate], an inhibitor of human carbonic anhydrase isozyme II [CA2] protein, is in Phase IV as a part of the combination drug quetiapine and topiramate as a treatment for alcohol abuse and alcohol use disorder	[110,113]
*DRD1*	Quetiapine, an antagonist of human DRD1 protein, is in Phase IV in combination with topiramate as a treatment for alcohol abuse	[113]
	SEROQUEL [quetiapine], a blocker of human DRD1 protein, is in Phase IV as a treatment for alcohol abuse	[114]
*DRD2*	SEROQUEL XR [quetiapine], a blocker of DRD2 protein, is in Phase IV in combination with topiramate as a treatment for alcohol abuse	[113]
	Quetiapine fumarate [quetiapine], an antagonist of human DRD2 protein, is in Phase IV as a treatment for alcohol abuse	[108]
	Risperidone, an antagonist of human DRD2 protein, is in Phase IV as a treatment for alcohol abuse	[109]
	Aripiprazole, an agonist of DRD2 protein, is in Phase III as a treatment for alcohol abuse	[111]
*DRD3, DRD4*	Aripiprazole, an agonist of DRD3, DRD4 proteins, is in Phase III as a treatment for alcohol abuse	[111]
*GABRA 1*	Topiramate, a modulator of human GABRA1 protein, is in Phase IV as treatment for alcohol abuse	[114]
	Acamprosate, a modulator of human GABRA1 protein, is in Phase IV as a part of the combination drug acamprosate and escitalopram as components of a treatment for alcohol abuse	[113]
*GRIN1, GRIN2B, GRIN2A, GRIN2C, GRIN2D, GRIN3A, GRIN3B*	Ketamine, an antagonist of human GRIN1, GRIN2B, GRIN2A, GRIN2C, GRIN2D, GRIN3A, and GRIN3B proteins, is in Phase III as a treatment for alcohol abuse	[115]
*HRH1, HTR2, HTR2B, HTR2C*	Risperidone and quetiapine, antagonists of human HRH1, HTR2, HTR2B, and HTR2C proteins, is in Phase IV as a treatment for alcohol abuse	[94, 108–109]
*HTR1A, HTR2A, HTR2C*	Quetiapine, an antagonist of human HTR1A, HTR2A, and HTR2C proteins, is in Phase III/IV as a treatment for alcohol abuse	[94, 111]
	Aripiprazole, an antagonist of human HTR1A, HTR2A, and HTR2C proteins, is in Phase III/IV as a treatment for alcohol abuse	[111]
*OPRD1, OPRM1, OPRK1, SIGMAR1*	Naltrexone, an antagonist of human OPRD1, OPRM1, OPRK1, and SIGMAR1 proteins, is in Phase III as a component of a treatment for alcohol abuse	[100–101]

**Table 3:** Genes reported related to alcohol dependence.

Gene symbol	Finding	Citations
*CA1, CA3, CA5A, CA5B, CA6, CA9*	Topiramate, an inhibitor of human CA1 protein, is in Phase IV as a treatment for alcohol dependence, along and as a part of the combination drug quetiapine and topiramate	[113, 116–118]
	Topiramate, an inhibitor of human CA6 protein, is in Phase III as a part of the combination drug for cognitive behavioral therapy	[119]
*GABRA1, GABRA3, GABRA4, GABRA5, GABRA6, GABRB2, GABRB3, GABRE, GABRG1, GABRG3, GABRP*	Olanzapine, an antagonist of human GABRA1, GABRA3, GABRA4, GABRA5, GABRA6, GABRB2, GABRB3, GABRE, GABRG1, GABRG3, and GABRP proteins, is in Phase III as a treatment for alcohol dependence	[120]
*GABRB1*	Gamma hydroxybutyric acid [4-hydroxybutanoic acid], an agonist of human GABRB1 protein, is in Phase IV as a treatment for alcohol dependence	[121]
	Olanzapine, an antagonist of human GABRB1 protein, is in Phase III as a treatment for alcohol dependence	[120]
*GABRG2*	Flumazenil, an antagonist of human GABRG2 protein, is in Phase II/III as a part of the combination drug flumazenil and gabapentin as a treatment for alcohol dependence	[122]
	Olanzapine, an antagonist of human GABRG2 protein, is in Phase III as a treatment for alcohol dependence	[120]
*GAD2*	Valproic acid, an inhibitor of human GAD2 protein, is in Phase IV in combination with lorazepam to prevent alcohol dependence	[123]
	Divalproex(valproic acid), an inhibitor of human GAD2 protein, is in Phase III in combination with lithium and quetiapine fumarate as a treatment for alcohol dependence	[124]
*OPRM1*	Mutant human MOR [*OPRM1*] gene (c.118 A>G) is associated with alcohol dependence in humans (p=0.0074).	[125]
*SLC6A4*	Escitalopram, an inhibitor of human SLC6A4 protein, is in Phase IV in combination with acamprosate as components of a treatment for alcohol dependence	[114]
	Fluoxetine, an inhibitor of human SLC6A4 protein, is in Phase IV in combination with naltrexone as a treatment for alcohol dependence	[126]
	Brisdelle [paroxetine], an inhibitor of human serotonin transporter [*SLC6A4*] protein, is in Phase IV as a treatment for alcohol dependence	[127]

**Table 4:** Genes reported related to Opioid Use Disorder (OUD).

Gene symbol	Finding	Citations
*ADRA2A*	Lofexidine and dexmedetomidine, agonists of human ADRA2A protein, are in Phase III as treatments for opioid-related disorders. Lofexidine is especially used in research on opiate dependence treatment	[128]
*ADRA2B, ADRA2C*	Dexmedetomidine, an agonist of human ADRA2B and ADRA2C proteins, is in Phase III as a treatment for opioid-related disorders	[129]
*CA1, CA2, CA3, CA4, CA5A, CA5B, CA6, CA7, CA9*	Acetazolamide, an inhibitor of human CA1, CA2, and CA3 proteins, is in Phase IV to prevent opioid-related disorders	[130]
*SLC6A2, SLC6A4*	Tramadol, an inhibitor of human SLC6A2 protein, is in Phase III as a treatment for opioid-related disorders	[131]

**Table 5:** Genes reported related to opioid dependence.

Gene symbol	Finding	Citations
*ABAT, ALDH5A1*	Valproic acid, an inhibitor of the ABAT protein and ALDH5A1 protein, is in Phase IV to treat opiate dependence	[93]
*ADRA1A, ADRA1B, ADRA1D, ADRA2A, ADRA2B, ADRA2C*	Paliperidone, an antagonist of ADRA1A, ADRA1B, ADRA1D, ADRA2A, ADRA2B, and ADRA2C proteins, is in Phase III to treat heroin dependence	[132]
*CACNA2D1, CACNA2D2*	Gabapentin, an inhibitor of human CACNA2D1 and CACNA2D2 proteins, is in Phase IV as a treatment for opioid dependence	[133]
*DRD2*	Buspirone, an agonist of human DRD2 protein, is in Phase IV as a treatment for heroin dependence	[103]
*DRD2, DRD3, DRD4*	Paliperidone, an antagonist of human DRD2, DRD3, and DRD4 proteins, is in Phase III as a treatment for heroin dependence	[132]
*GABRA1, GABRA3, GABRA4, GABRA5, GABRA6, GABRB1, GABRB2, GABRE, GABRG1, GABRG2, GABRG3, GABRP*	Isoniazid, an inhibitor of human GABRA1, GABRA3, GABRA4, GABRA5, GABRA6, GABRB1, GABRB2, GABRE, GABRG1, GABRG2, GABRG3, and GABRP proteins, is in Phase IV in combination with naltrexone as a treatment for opioid dependence	[134–135]
*GABRB3*	Isoniazid, an inhibitor of human GABRB3 protein, is in Phase IV in combination with naltrexone as a treatment for opioid dependence	[134–135]
	Mutant human *GABRB3* (SNP substitution [rs7165224]) is associated with heroin addiction in humans (p=0.01).	[136]
*GAD2*	Valproic acid, an inhibitor of human GAD2 protein, is in Phase IV in combination with buprenorphine as a treatment for opiate dependence	[89]
*GRIN1, GRIN2B, GRIN2A, GRIN2C, GRIN2D, GRIN3A, GRIN3B*	Ketamine, an antagonist of human GRIN1, GRIN2B, GRIN2A, GRIN2C, GRIN2D, GRIN3A, and GRIN3B proteins, is in Phase IV as a treatment for opiate dependence	[137]
	Memantine, an antagonist of human GRIN1, GRIN2A, GRIN2B, GRIN2C, GRIN2D, GRIN3A, and GRIN3B proteins, is in Phase II/III as a treatment for opioid dependence	[138]
	Memantine, an antagonist of human GRIN1, GRIN2A, GRIN2B, GRIN2C, GRIN2D, GRIN3A, and GRIN3B proteins, is being tested in combination with Vivitrol [naltrexone] as a treatment for opioid dependence	[139]
*GRIN3A, HTR3A*	Methadone, an antagonist of human GRIN3A and HTR3A proteins and mu-opioid receptor, has been approved as a treatment for opioid dependence	[140]
*HRH1*	Mirtazapine, a blocker of human HRH1, HTR2A, HTR2B, and HTR2C proteins, is in Phase III as a treatment for heroin dependence	[141]
	Paliperidone, an antagonist of human HRH1, HTR2A, and HTR2C proteins, is in Phase III as a treatment for heroin dependence	[142]
*HTR1A*	Buspirone, an agonist of human HTR1A proteins, is in Phase IV as a treatment for heroin dependence	[103]
*HTR2A, HTR2C*	Mirtazapine, a blocker of human HRH1, HTR2, HTR2A, HTR2B, and HTR2C proteins, is in Phase III as a treatment for heroin dependence	[141]
	Paliperidone, an antagonist of human HRH1, HTR2A, and HTR2C proteins, is in Phase III as a treatment for heroin dependence	[142]
	Buspirone, an agonist of human HTR1A, HTR2A, HTR2B, and HTR2C proteins, is in Phase IV as a treatment for heroin dependence	[103]
*HTR2B,*	Mirtazapine, a blocker of human HRH1, HTR2, HTR2A, HTR2B, and HTR2C proteins, is in Phase III as a treatment for heroin dependence	[141]
	Buspirone, an agonist of human HTR1A, HTR2A, HTR2B, and HTR2C proteins, is in Phase IV as a treatment for heroin dependence	[103]
*OPRM1*	Mutant human *OPRM1* (c.118A>G [germline] (rs1799971)) is observed to carry susceptibility to opioid dependence type 1 in humans	[55]
	Naloxone, an antagonist of human OPRM1 protein, has been approved in the combination Cassipa [buprenorphine/naloxone] (maintenance) as a treatment for opioid dependence	
	Naltrexone, an antagonist of human OPRM1 protein, is in Phase IV as a treatment for opioid dependence	[56, 143]
	Buprenorphine, antagonist of OPRM1 protein, and naloxone in combination are in Phase IV as a treatment for opioid dependence.	[144]
	Hydromorphone, an agonist of human OPRM1 protein, is in Phase III as a component of a treatment for opioid dependence	[145–146]
	Buprenorphine, an antagonist of OPRM1 protein, and naloxone in combination are in Phase III as a treatment for heroin dependence	[147–155]
*OPRD1, OPRK1*	Naloxone, an antagonist of human OPRD1 and OPRK1 proteins, has been approved as a part of the combination drug Cassipa [buprenorphine/naloxone] (maintenance) as a treatment for opioid dependence	[147]
	Naltrexone, an antagonist of human OPRD1 and OPRK1 proteins, is in Phase IV as a treatment for opioid dependence	[56, 143]
	Buprenorphine, an agonist of human OPRD1 and antagonist of OPRK1 proteins, and naloxone in combination are in Phase IV as a treatment for opioid dependence	[144]
	Hydromorphone, an agonist of human OPRD1 and OPRK1 proteins, is in Phase III as a component of a treatment for opioid dependence	[145–146]
	Buprenorphine, agonist of human OPRD1 and antagonist of OPRK1 protein, and naloxone and a combination are in Phase III as a treatment for heroin dependence	[147–155]
*SIGMAR1*	Naloxone, an antagonist of human SIGMAR1 protein, has been approved as a part of the combination drug Cassipa [buprenorphine/naloxone] (maintenance) as a treatment for opioid dependence	[147]
	Naltrexone, an antagonist of human SIGMAR1 protein, is in Phase IV as a treatment for opioid dependence	[56, 143]
	Hydromorphone, an agonist of human SIGMAR1 protein, is in Phase III as a component of a treatment for opioid dependence	[145–146]
*SLC6A2, SLC6A3, SLC6A4*	Bupropion, an inhibitor of human SLC6A2, SLC6A3, and SLC6A4 proteins, is in Phase III as a component of a treatment for opioid dependence	[156]

**Table 6:** Genes reported related to opioid addiction.

Gene symbol	Finding	Citations
*ADRA1A*	The SNP substitution (rs486179) mutation in human *ADRA1* leads to heroin addiction in humans	[136]
*DRD1*	Mutant human *DRD1* (SNP substitution [rs5326]) is associated with heroin addiction in humans (p=0.01)	[136]
*GABRB3*	Mutant human *GABRB3* (SNP substitution [rs7165224]) is associated with heroin addiction in humans (p=0.01).	[136]
*GAD 2*	Mutant human *GAD2* (SNP substitution [rs8190646]) is associated with heroin addiction in humans (p=0.01).	[136]
*GRIN2A*	Mutant human *GRIN2A* is associated with heroin addiction in humans	[136]
*GRIN3A, HTR3A*	Methadone, an antagonist of human GRIN3A, HTR3A protein, and mu-opioid receptor, has been approved as a treatment for opioid addiction	[140]
*OPRD1, OPRM1, OPRK1*	Methadone, an agonist of human OPRD1 protein, has been approved as a treatment for opioid addiction	[140]
